# Therapeutic Inducers of Apoptosis in Ovarian Cancer

**DOI:** 10.3390/cancers11111786

**Published:** 2019-11-13

**Authors:** Mudra Binju, Monica Angelica Amaya-Padilla, Graeme Wan, Hendra Gunosewoyo, Yohan Suryo Rahmanto, Yu Yu

**Affiliations:** 1 School of Pharmacy & Biomedical Sciences, Curtin University, Curtin Health Innovative Research Institute, Perth, WA 6102, Australia; 2 Sidney Kimmel Comprehensive Cancer Center, Johns Hopkins Medical Institutions, Baltimore, MD 21231, USA; 3 Department of Pathology, Johns Hopkins Medical Institutions, Baltimore, MD 21205, USA; 4 University of Western Australia Medical School, Division of Obstetrics & Gynaecology, Perth, WA 6009, Australia

**Keywords:** ovarian cancer, platinum-resistance, apoptosis proteins

## Abstract

Ovarian cancers remain one of the most common causes of gynecologic cancer-related death in women worldwide. The standard treatment comprises platinum-based chemotherapy, and most tumors develop resistance to therapeutic drugs. One mechanism of developing drug resistance is alterations of molecules involved in apoptosis, ultimately assisting in the cells’ capability to evade death. Thus, there is a need to focus on identifying potential drugs that restore apoptosis in cancer cells. Here, we discuss the major inducers of apoptosis mediated through various mechanisms and their usefulness as potential future treatment options for ovarian cancer. Broadly, they can target the apoptotic pathways directly or affect apoptosis indirectly through major cancer-pathways in cells. The direct apoptotic targets include the Bcl-2 family of proteins and the inhibitor of apoptotic proteins (IAPs). However, indirect targets include processes related to homologous recombination DNA repair, micro-RNA, and *p53* mutation. Besides, apoptosis inducers may also disturb major pathways converging into apoptotic signals including janus kinase (JAK)/signal transducer and activator of transcription 3 (STAT3), wingless-related integration site (Wnt)/β-Catenin, mesenchymal-epithelial transition factor (MET)/hepatocyte growth factor (HGF), mitogen-activated protein kinase (MAPK)/extracellular signal-regulated kinase (ERK), and phosphatidylinositol 3-kinase (PI3K)/v-AKT murine thymoma viral oncogene homologue (AKT)/mammalian target of rapamycin (mTOR) pathways. Several drugs in our review are undergoing clinical trials, for example, birinapant, DEBIO-1143, Alisertib, and other small molecules are in preclinical investigations showing promising results in combination with chemotherapy. Molecules that exhibit better efficacy in the treatment of chemo-resistant cancer cells are of interest but require more extensive preclinical and clinical evaluation.

## 1. Apoptosis in Ovarian Cancer

Ovarian cancer (OC) is one of the most aggressive types gynecologic cancer worldwide with a five-year survival rate of around 30–40% of the patients diagnosed with high grade serous ovarian carcinoma (HGSOC) [1,2]. Despite various developments in effective treatment strategies, survival outcomes barely improved over the past decade [3,4]. Most patients who receive standard treatment relapse within 6–12 months after completing chemotherapy [5]. Studies have shown that the mechanisms underlying platinum-resistance are genetic mutation, epigenetic alteration, evasion of apoptosis, and other external environmental factors [4,5]. Hence, there is a need to develop a treatment strategy to combat OC.

Apoptosis, also known as programmed cell death, is initiated by two main pathways: the intrinsic or mitochondrial-mediated pathway and extrinsic or the death-receptor pathway [6,7]. The intrinsic pathway is induced by various external factors such as ultraviolet (UV)- or gamma- radiation, DNA-damage, oncogenic factors, and viral infections [8]. The stress signals received by intercellular molecules mediate mitochondrial outer membrane permeabilization (MOMP) and release pro-apoptotic molecules into the cytoplasm [8]. Two of the main components of the intrinsic apoptotic pathway are B-cell lymphoma-2 (BCL-2) family and inhibitors of apoptotic proteins (IAPs) that serve as an apoptotic switch, controlling the permeabilization of mitochondrial membrane [9]. The extrinsic pathway, on the other hand, is initiated by the association of cell surface receptors, a subset of tumor necrosis factor (TNF) with their respective activating cytokine ligands from TNF-superfamily of proteins [10]. Some suggest that these two pathways may work in synchronization to start apoptosis [11]. Despite conserved apoptosis processes, cancer cells develop mechanisms to inhibit apoptosis by altering anti-apoptotic molecules or inactivating pro-apoptotic cell death components [11]. Cancer cells have adopted several mechanisms to evade apoptosis [6,12,13,14]. These mechanisms can be tumor-type specific or regularly altered across a different range of tumors [11]. Thus, current research efforts focus on identifying of the molecules capable of restoring apoptosis in cancer cells without affecting normal cells nearby [6]. In this paper, we discuss selected molecules with less well-known anti-apoptotic mechanisms in OC. Targeting these mechanisms may promise in restoring apoptosis or sensitizing chemo-resistance ovarian cancer cells.

## 2. Direct Targets in Apoptotic Pathways

### 2.1. BCL-Protein Inhibitors

**Figure 1:**
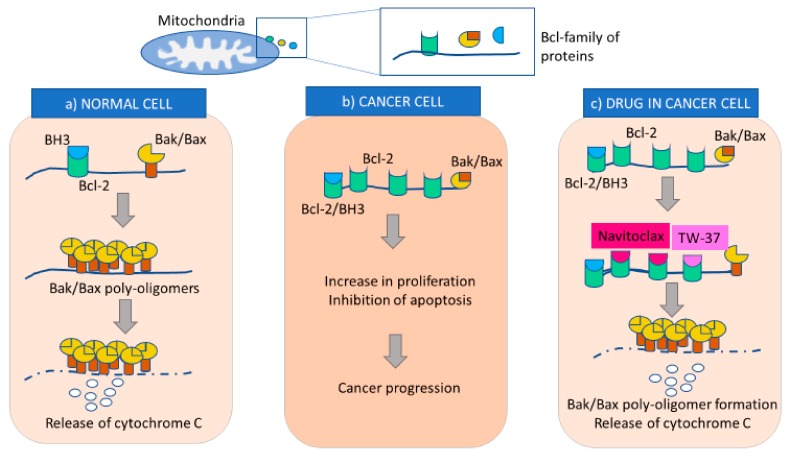
Bcl-protein dependent apoptotic pathway in a normal cell, cancer cell and a cancer cell treated with apoptotic inducers. (**a**) In a normal cell, Bcl-2 acts as an inhibitor of apoptosis and Bcl-2 homologous antagonist killer (Bak)/B-cell associated X protein (Bax) complex initiates apoptosis upon stress. Bcl-2 homology domain 3 (BH3) forms a complex with Bcl-2 to deactivate Bcl-2 and Bax/Bak can initiate apoptosis by forming symmetric poly-oligomers which causes mitochondrial outer membrane permeabilization (MOMP) and releases cytochrome C in the cytoplasm for initiation of apoptosis. (**b**) However, in cancer cell, Bcl-2 is over-expressed and the concentration of BH3 is comparatively low. Thus, Bcl-2 is upregulated resulting in inhibition of apoptosis. (**c**) Navitoclax and TW-37 mimic BH3 and form a complex with Bcl-2 to deactivate proliferation and initiate apoptosis by Bak/Bax protein complex formation.

Bcl-2 family of proteins plays a vital role in regulating the intrinsic mitochondrial pathway of apoptosis [15]. They classify into three subgroups depending on their function: anti-apoptotic, pro-apoptotic multi-domain effectors, and pro-apoptotic Bcl-2-homology (BH)3-only proteins [16]. The anti-apoptotic subgroup consists of Bcl-2, Bcl extra-large (Bcl-XL), myeloid cell leukemia 1 (Mcl-1), Bcl-2-like 2 (Bcl-w), Bcl-2 homologue-B (Bcl-B), and Bcl-2-related protein A1 (Bcl-2A1, or A1) [17,18]. These proteins promote cell survival by sequentially inhibiting pro-apoptotic Bcl-2-family of proteins [19]. The pro-apoptotic proteins B-cell associated X protein (Bax) and Bcl-2 homologous antagonist killer (Bak) and Bcl-2 related ovarian killer (BOK) contain BH-domain 1 to 4 [20]. Bak and Bax are initiators of apoptosis by forming an oligomer on the outer mitochondrial membrane (OMM) that leads to the MOMP [21]. Anti-apoptotic proteins interact with pro-apoptotic Bcl-2 homology domain 3 (BH3) only proteins via BH-domains by forming a hydrophobic cleft [22]. Recently, reports have shown that other domains, such as the transmembrane domain of Bcl-2 and Bcl-xL can also interact with the transmembrane domain of Bax to initiate apoptosis [23]. Interestingly, BH4 domain of Bcl-2 promotes anti-apoptotic activity by binding to inactive Bax [24]. Studies have shown that cancer cells express higher levels of BH3-only proteins, Bcl-2-like protein 11 (Bim) and BH3 interacting domain death agonist (Bid) [25]. Additionally, cancer cells mediate pro-survival mechanisms by anti-apoptotic Bcl-2 family members, allowing for tumor progression [26,27,28]. Bcl-2 has also been identified in ovarian cancer cells to promote survival and drug resistance [29]. Thus, targeting the interaction of these Bcl-proteins may promote apoptosis in cancer cells (Figure 1).

Navitoclax (also known as ABT-263), an orally bioavailable analog of ABT-737, is one of the first BH3 mimetics identified for cancer therapy. Navitoclax is a known inhibitor of Bcl-2, Bcl-XL, and Bcl-w, crucial for Bcl-mediated pathway. This inhibitor mimics the BH3 domain interaction of Bad proteins with Bcl-2 family of proteins causing apoptosis by promoting the activities of Bax, Bak and BH3-only proteins [30]. A study by Wong et al. examined the effects of combining navitoclax with chemotherapy in 27 ovarian cancer cell lines and showed promising synergistic effect [31]. The higher levels of Bcl-XL correlated to the poor chemotherapy response in ovarian cancer patients [31]. Another study examined the effects of this molecule in combination with chemotherapeutic agents in drug-resistant ovarian cancer cells in vitro and in xenograft models [32]. In this study, navitoclax alone inhibited the growth of eight ovarian cancer cell lines with low potency but sensitized the cells to carboplatin cytotoxicity by inducing more rapid apoptosis. Navitoclax also increased carboplatin sensitivity in one of three primary cultures derived from ascites. Later, other studies suggested that phosphatidylinositol 3-kinase (PI3K) pathway inhibitor (pictilisib) and navitoclax combination treatment resulted in enhanced pitavastatin-induced apoptosis in ovarian cancer cells [33]. This evidence suggests that Navitoclax is able to enhance the efficacy of chemotherapy drugs in ovarian cancer cells. In addition, it will be interesting to test the synergy of Navitoclax with other drugs, for example, PI3K inhibitors. There is also a need to test this drug in patient-derived cells or xenograft models to predict the response and toxicity of these drugs in patient cells before advancing to clinical trials.

A small-molecule inhibitor, TW-37, is gaining attention because of its significant inhibitory action against Bcl-2. TW-37 binds to the BH3 domain inhibiting pro-apoptotic proteins such as Bid, Bim, and Bad [34]. A study examined the molecular mechanism of TW-37 in enhancing cisplatin-induced apoptosis in ovarian cancer cells [35]. The ovarian cancer cell lines: SKOV3, OVCAR3, OV-90, and 3AO and cisplatin-resistant cell line–SKOV3^DDP^ were used; and Bcl-2 expression levels were the highest in SKOV3^DDP^. Furthermore, cell viability experiments showed a significant decline in cell growth after treatment with combined TW-37 and cisplatin in cell lines with high Bcl-2 levels. The treated cells also exhibited an increase in apoptosis showing that TW-37 sensitizes cells to cisplatin as confirmed via enzyme-linked immunosorbent assay (ELISA) and terminal deoxynucleotidyl transferase dUTP nick end labeling (TUNEL) assays [35]. TW-37 treatment also decreased colony formation as compared to untreated cells. Although results show that TW-37 may be a useful drug candidate for treating cisplatin-resistant ovarian cancer cells, cell lines used in this study are not a reliable model to imitate the patient’s cell response. Hence, it will be interesting to test this molecule using xenograft models or patient-derived cancer cells

### 2.2. IAPs Inhibitors

Synergy between the activators and inhibitors of apoptosis can alter chemotherapy-induced apoptosis in cancer cells, resulting in innate or acquired chemotherapy resistance [36,37]. The mechanism of platinum-induced apoptosis involves the release of cytochrome c, SMAC (second mitochondria-derived activator of caspases) and tumor necrosis factor (TNF)-alpha [38]. One approach to induce apoptosis is the use of small-molecule drugs mimicking the pro-apoptotic mitochondrial protein, SMAC, which binds to IAPs [39]. Although there are eight members of IAPs in mammals, the most commonly investigated IAPs are cIAP1 and cIAP2. These proteins communicate with tumor necrosis factor receptor-associated factor 2 (TRAF2) to block caspase 8 activation complex, resulting in the inhibition of TNF-induced apoptosis [40,41,42]. Similarly, X-linked IAP (XIAP) antagonizes three caspases–caspase 3 and 7 (effectors of apoptosis), caspase 9 (initiator of apoptosis), which blocks intrinsic and extrinsic apoptosis [40,42,43]. Various reports have emerged claiming that IAPs are deregulated in ovarian cancer cells and XIAP are involved in ovarian cancer chemoresistance [44,45,46,47]. Thus, the inhibition of XIAP is a mechanism leading to increased apoptosis in platinum-resistant ovarian cancer cells in vitro and in vivo [45,47,48]. In mice models, XIAP inhibition reduced tumor progression and increased animal life expectancy [49,50].

**Figure 2:**
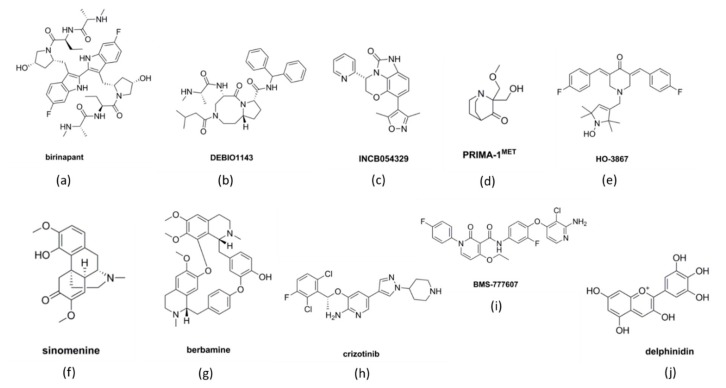
Chemical structure representation of molecules identified as inducers of apoptosis. (**a**,**b**) Inhibitors of inhibitors of apoptotic proteins (IAPs), birinapant and DEBIO1143, (**c**) inhibitor of homologous recombination (HR), INCB054329 (**d**) *p53* effector, PRIMA-1^MET^ (**e**) janus kinase (JAK)/signal transducer and activator of transcription 3 (STAT3) pathway inhibitor, HO-3867 (**f**,**g**) wingless-related integration site (WNT)/β-catenin pathway inhibitor, Sinomenine and berbamine; (**h**,**i**) mesenchymal-epithelial transition factor (MET)/hepatocyte growth factor receptor (HGF) pathway inhibitor, crizotinib and BMS-777607; (**j**) mitogen-activated protein kinase (MAPK)/extracellular signal-regulated kinase (ERK) pathway inhibitor, delphinidin.

Birinapant (Figure 2a) is a small molecule protein-like chain of SMAC and inhibitor of IAP. Janzen et al. showed that birinapant suppresses IAPs in combination with carboplatin [51]. Birinapant combined with carboplatin significantly increased tumor regression in a xenograft model [51]. Therefore, birinapant is a promising candidate and has entered clinical trials (ClinicalTrial.gov identifiers: NCT01681368 and NCT01940172) for advanced platinum-resistant or relapsed epithelial ovarian cancer (EOC) (Table 1). More recent data suggest that birinapant is suitable for improving carboplatin sensitivity in platinum-resistant tumors with high levels of cellular IAP (cIAP) [52]. These observations further suggest that it is possible to promote apoptosis in platinum-resistant settings.

APG-1387 is another SMAC mimetic that has been found effective against ovarian cancer cells [53]. Cell viability assay showed a significant decline in cell survival in ovarian cancer cells upon treatment with APG-1387 as compared to control. Further investigation disclosed that this molecule activates caspase-mediated apoptosis in a tumor xenograft model.

DEBIO-1143 (AT-406 or SM-406; Figure 2b) is another SMAC mimetic that has been studied with the intention to improve platinum-induced apoptosis in ovarian cancer cells [54,55]. Studies showed that the use of DEBIO-1143 in combination with carboplatin was beneficial in sensitizing carboplatin-resistant ovarian cancer cells within an in vivo mouse model and SKOV3 ovarian cancer cell line [54]. DEBIO-1143 antagonize multiple IAPs and have been claimed to have anti-tumorigenic activity against at least 60% of the carboplatin-resistant ovarian cancer cell lines [56,57]. DEBIO-1143 promoted XIAP degradation and initiated poly-ADP ribose polymerase (PARP)-dependent apoptotic pathway displaying single-agent activity against ovarian cancer cells [56]. Recently, DEBIO-1143 entered clinical trials (ClinicalTrials.gov Identifier: NCT01930292) for the treatment of patients with either breast, lung, or ovarian cancer in combination with carboplatin and paclitaxel (Table 1) [56,57,58]. Patients were advised of the 50–400 mg dose until the recommended dose could be set for five consecutive days in a 21-day treatment cycle. However, the study was terminated earlier than planned. Thus, there is an immense need to study the safety of SMAC mimetics. 

## 3. Indirect Cellular Processes that Affect Apoptosis Induction

### 3.1. Homologous Recombination

Homologous recombination (HR) is a DNA repair mechanism for double-stranded breaks. Upon DNA damage, proteins such as poly (ADP-ribose) polymerase-1 (PARP1), breast cancer type 1 (BRCA1) and breast cancer type 2 (BRCA2) are activated to initiate the repair mechanism [59,60]. Cancer cells evade apoptosis caused by chemotherapy by altering this process. Hence, to overcome this issue, inhibitors of HR were developed to inhibit the cell from repairing DNA damage caused by chemotherapy. In HGSOC, HR-proficient tumors have poorer clinical outcomes to the standard platinum-based therapy [60] and display reduced response to poly-ADP ribose polymerase inhibitors (PARPi) [61,62]. However, loss of functional BRCA proteins causes the failure of the repair mechanism, leading to apoptosis [60]. Ovarian cancer patients carrying BRCA mutation display advanced grade of disease and are found highly sensitive to PARPi and platinum drugs [61]. However, platinum sensitivity is not a true indicator of PARPi response. Almost 50% of the high grade serous ovarian carcinoma (HGSOC) are HR deficient, out of which, 20% are due to mutations in BRCA1/2 that affect the PARPi sensitivity in HGSOC [62]. Thus, a 60-gene panel known as BRCAness profile has been generated that helps in identifying BRCA-mutated ovarian tumors. This tool has been effective in identifying PARPi response accurately in 80% of the ovarian tumor [63]. Following the success of PARPi, the Food and Drug Administration (FDA) has approved Olaparib, Rucaparib, and Niraparib, as maintenance therapy in ovarian cancer treatment. However, ovarian cancer cells may restore BRCA functionality to develop resistance against PARPi [64,65]. Therefore, there is an unmet need to identify new molecular targets to treat HR-proficient tumors.

Wilson et al. identified that HR-proficient tumors showed amplification of bromodomain and extra-terminal domain (BET) genes such as *BRD4*, that recognize lysine residues on histone tails to promote gene transcription via epigenetic regulation [66]. According to the Cancer Genome Atlas database, expression of *BRD4* is amplified in almost 10% of the HGSOC [67]. BRD proteins interact with acetylated lysine residues via bromodomain to initiate transcription. Therefore, targeting BRD4 in ovarian cancer cells with its elevated expression should sensitize the cells to PARPi [68,69]. A study has identified INCB054329 (Figure 2c) as a BET inhibitor [61]. Preclinical testing in vivo (patient-derived xenograft, PDX) and in vitro (EOC cells—SKOV3, OVCAR3, OVCAR4, UWB1.289+BRCA1 wild type (BRCA1 WT) and UWB1.289 BRCA1 null (BRCA1 Null)) models showed that INCB054329 sensitized the cells to PARPi reducing cell growth, increasing DNA damage and apoptosis in the HR-proficient ovarian cancer cells [70]. Therefore, these data suggest that apoptosis can be induced by altering DNA repair mechanisms.

### 3.2. p53 Mutation

*TP53* is the most common mutation found in almost 96% of HGSOC cases [62,71,72,73]. *TP53* is located on chromosome 17p, encoding pro-apoptotic protein p53 which similarly plays a critical role as a tumor-suppressor [74]. The p53 protein plays a critical role in Bcl-mediated apoptosis. This protein regulates pro-apoptotic BH3-only proteins—PUMA and NOXA—to induce apoptosis [75,76]. Additionally, other components of Bcl-2 regulated pathway–Bax and Apaf-1 are also regulated by p53 [77]. However, mutations in p53 alter the tumor suppressive capabilities and promote oncogenic properties [78,79]. Studies suggest that p53 mutation can be a prognostic marker to detect the aggressiveness and platinum response of tumor at an early stage [80]. Anticancer agents induce apoptosis in ovarian cancer cells by damaging DNA in dividing cells. Under such stress conditions, normal cells respond by increasing the expression of p53 [81]. Following this, the cell can either initiate apoptosis due to DNA damage or enter cell cycle arrest mode making them non-responsive to chemotherapy [82]. However, in the case of p53 mutation or absence, the cell is unable to follow either of these pathways and undergoes continuous proliferation [82]. Thus, several agents have been designed to preserve normal p53 functionality. PRIMA-1 (p53 reactivation and induction of massive apoptosis; Figure 2d) and its methylated form PRIMA-1^MET^ have recently emerged as molecules to reverse p53 mutation to wild-type p53 in various cancers such as breast, neck, thyroid, and melanoma [83,84,85,86]. PRIMA-1^MET^ displays more promising results when compared to the unmethylated form and has entered clinical trials to evaluate efficacy in refractory hematologic malignancies and prostate cancer (Table 1) [87]. A study investigated how PRIMA-1^MET^ induced apoptosis via the p53 mechanism and suggested a mechanism involving reactive oxygen species (ROS) [88]. The results showed that PRIMA-1^MET^ inhibited antioxidant enzymes, such as Prx3 and GPx-1, ultimately leading to apoptosis. Altogether, it was evident that PRIMA-1^MET^ exhibits anti-tumor activity via the accumulation of ROS irrespective of p53 mutation status in the EOC [88]. Although PRIMA-1^MET^ shows promising results as a novel therapeutic target, its suitability in ovarian cancer treatment requires more detailed preclinical analyses.

### 3.3. Micro-RNAs in Inducing Apoptosis

Micro-RNAs (miRNA) are a class of non-coding RNAs that regulate gene expression at the post-transcriptional level by binding to the 3’ untranslated region of mRNA thereby causing degradation of mRNA [89]. The role of miRNA has been identified in various crucial cellular processes such as cell growth, differentiation, and death [90,91]. In cancer cells, miRNAs levels can be altered and have roles to either promote cancer progression or act as tumor suppressors. For example, down-regulation of *let-7* in lung cancer may promote an increase in *RAS* and *HMGA2* oncogene expression and deletion or down-regulation of *mir-15* and *mir-16* in leukemia reduced *Bcl-2* expression mediated apoptosis [92,93,94,95]. Studies have shown that miRNA is also involved in ovarian carcinogenesis [96]. Specifically, the expression of several miRNAs including *miR-199a*, *miR-200a*, *miR-200b*, and *miR-200c* was over-expressed, whereas, *miR-140*, *miR-145*, and *miR-125b1* were downregulated compared to normal tissues [97]. In addition, *Let-7a-2* and the *miR-200* family of micro-RNA were consistently downregulated in ovarian carcinoma [96]. These reports along with many others have compelled research into miRNAs for the treatment of ovarian cancer. Many studies have reported promising results whereby miRNAs targeting can affect apoptotic regulation.

The *miR-124* is a tumor suppressor and is often downregulated in a variety of cancers [97,98,99,100]. *miR-124* has been shown to target the epithelial–mesenchymal transition (EMT), angiogenesis, and cell proliferation [101,102,103,104,105,106]. In ovarian cancer, *miR-124* is downregulated and has been identified to inhibit cancer progression by multiple mechanisms, including the regulation of cell migration and invasion through Sphingosine kinase 1 (SphK1) [107,108,109] and cell proliferation, cell death, and apoptosis by targeting programmed cell death 6 (PDCD6), a pro-apoptotic protein [110]. PDCD6 over-expression in cells co-transfected with *miR-124* reversed *miR-124*-induced apoptosis. In addition, *PDCD6* expression has been shown to regulate cell viability and tumor necrosis factor receptor 1-induced cell death [111,112]. Thus, the enhancement of *miR-124* expression can lead to apoptosis in epithelial ovarian cancer cells.

The family of Let-7 miRNAs are regulators of differentiation and apoptosis in eukaryotic cells [113,114,115]. The Let-7 family targets oncogenes that regulate cell growth and apoptosis in cancer cells; however, it is mostly found to be down-regulated in different types of cancers [116]. Reports have shown that restoring the original expression of *Let-7* miRNAs in cancer cells inhibits tumorigenesis [117]. *Let-7d-3p* miRNA has been associated with apoptosis and response to neoadjuvant chemotherapy in ovarian cancer [118]. The increased expression of *let-7d-3p* was correlated with a positive response to platinum/paclitaxel treatment in ovarian cancer patients. Additionally, *let-7d-5p* miRNA was reported to rescue ovarian cancer cells apoptosis and chemosensitivity by modulating the p53 pathway via high a mobility group 1 (HMGA1) [119].

**Figure 3:**
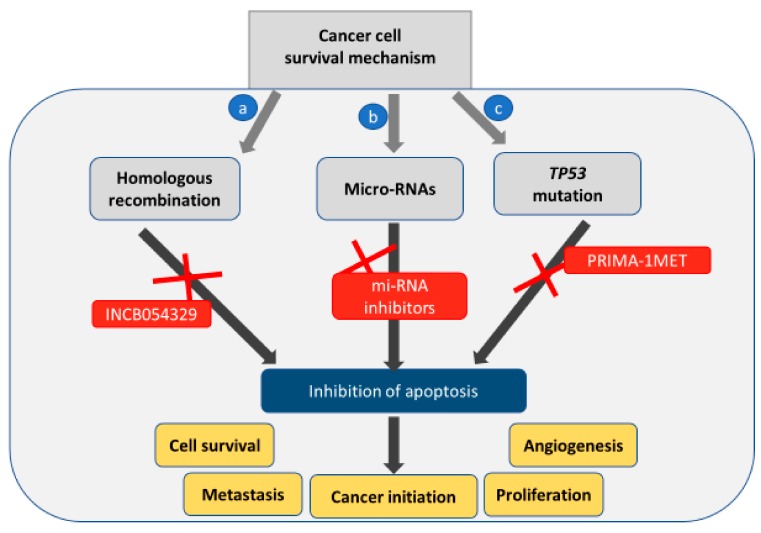
Indirect cellular processes that contribute to the inhibition of apoptosis in a cancer cell. (**a**) Homologous recombination (HR) repairs the damaged DNA induced by chemotherapy leading to inhibition of apoptosis. The use of HR inhibitors can lead to apoptosis. (**b**) micro-RNAs. Inhibiting of micro-RNAs by other micro-RNAs or their inhibitors can lead to apoptosis. (**c**) p53 mutation commonly found in cancer cells also inhibits apoptosis and promotes cancer cell survival. Therefore, PRIMA-1^MET^ restores the function of p53 protein and promotes cancer cell death.

The miRNA-200 family consists of five miRNAs members: *miR-200a*, *miR-200b*, *miR-200c*, *miR-141* and *miR-429* [120,121]. This set of miRNAs are regulators of EMT in neoplastic formations and in organs with high activity such as ovaries [122,123,124,125,126,127]. The association of miR-200 family with ovarian carcinoma was first investigated by Cao et al. [128]. This study recruited 100 patients with EOC and 50 healthy people to identify differential expression of miRNA levels. The results showed that the expression of miR-200 family was significantly higher in cancer patients as compared to healthy subjects. Moreover, the higher expression of *miR-200c* was exclusively associated with carcinoma at the advanced stages [128]. A study carried out by Liu and colleagues claimed that enhancing the expression of *miR-200b* and *miR-200c* could lead to apoptosis in EOC [129]. Results from this study indicated that *miR-200b* and *miR-200c* control the downregulation of DNA methyltransferase (DNMTs) and increase chemotherapeutic sensitivity in ovarian cancer cells leading to better drug efficacy and tumor suppression. Thus, solidifying the need to investigate the regulation of miR-200 family in suppressing ovarian cancer further. Therefore, apoptosis can be indirectly affected by changes in HR repair, p53 mutation and miRNA levels (Figure 3).

## 4. Targeting Cancer-Associated Pathways to Induce Apoptosis

### 4.1. JAK/STAT3 Pathway

Activation of Janus activated kinase 2 (JAK2)/signal transducer and activation of transcription-3 (STAT3) pathway is involved in cancer progression through the enhancement of cell proliferation, differentiation, and angiogenesis [130,131]. STAT3 is a transcription factor involved in the mediation of cytokines and growth factor signaling [132]. STAT3 has been reported to be over-expressed in cancer cells [133,134]. 

In ovarian cancers, STAT3 over-activation has been associated with a reduced platinum response and poor prognosis [134,135]. Rath et. al have shown that OCs express elevated levels of interleukin 6 (IL-6), an activator of the JAK2/STAT3 signaling pathway as compared to benign and normal tissues [136]. Additionally, high levels of IL-6 in ascites, serum, and malignant cells are used as a marker to predict overall patient’s survival [137,138,139,140]. These data suggest that JAK2/STAT3 pathway plays an important role in the progression and poor survival associated with OC. Thus, various studies are invested in finding an efficient drug to target this pathway and improve the platinum response in OC patients.

Curcumin analogues, such as HO-3867 (Figure 2e), which belong to the diarylidenyl piperidin-4-ones (DAP) family of anti-cancer agents that suppress cellular growth and metastasis by targeting STAT3 in ovarian cancer cells [141]. Several studies in the past decade have also examined the role of HO-3867 in inducing programmed cell death in ovarian cancer cells [142,143,144]. Rath et. al. claimed that a nitroxide precursor, *N-hydroxypyrroline* (-NOH) moiety present in HO-3867 is crucial for selective targeting of STAT3 in cancerous cells [145]. Two similar compounds from DAP family of drugs—HO-3867 (possessing -NOH moiety) and HO-4073 (lacking -NOH moiety) —were tested in cancerous and non-cancerous cells. Due to the difference in backbone conjugation with -NOH group, there was a change in the interaction of these compounds with STATs [145]. Using a tumour xenograft model and primary cisplatin-resistant and -sensitive cells, HO-3867 selectively targets STAT3 leading to apoptosis only in the cancerous cells without affecting the non-cancerous cells.

**Figure 4:**
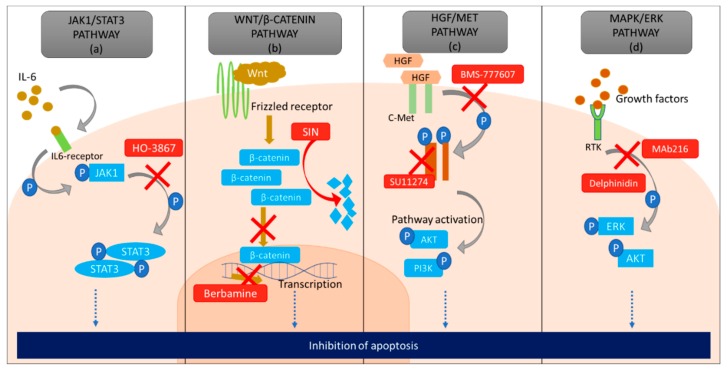
A diagrammatic representation of the pathways that can indirectly lead to inhibition of apoptosis in a cancer cell. (**a**) Janus activated kinase 1 (JAK1)/signal transducer and activation of transcription-3 (STAT3) pathway is upregulated due to the accumulation of interleukin 6 (IL-6) which causes phosphorylation of JAK1 and STAT3, promoting cell survival and increase proliferation. However, HO3867 inhibits phosphorylation of STAT3 causing apoptosis. (**b**) Wnt pathway is activated by the binding of Wnt molecule to the frizzled receptor (FZD). This causes the formation of a complex and accumulation of β-catenin in the cytoplasm. Saturation of β-catenin in cancer cells causes its translocation into the nucleus, where it initiates uninterrupted transcription leading to an increase in proliferation and chemoresistance. However, sinomenine (SIN) degrades β-catenin in the cytoplasm and berbamine inhibits transcription in the nucleus of cancer cells eventually causing apoptosis. (**c**) Hepatocyte growth factor (HGF) pathway initiates upon binding of HGF to c-Met (tyrosine kinase receptor) which leads to phosphorylation of c-Met, which in turn activates various other cell signaling pathways including AKT, PI3K pathways important for cell survival, proliferation and angiogenesis. BMS-777607 obstructs the phosphorylation of c-MET and SU11274 decreases the concentration of c-MET in the cell to inhibit HGF pathway, both leading to apoptosis (**d**) Growth factors attach to receptor tyrosine kinase (RTK) present on the extracellular surface of the cell and can switch on the mitogen-activated protein kinase (MAPK)/extracellular signal-regulated kinase (ERK) pathway in the cell. An increased concentration of growth factors leads to over-activation of MAPK/ERK pathway in cancer cells. Delphinidin and Mab216 down-regulates this pathway by inhibiting phosphorylation of ERK, AKT, which eventually leads to apoptosis.

Another study showed that the expression of STAT3 was significantly reduced in cells treated with HO-3867 and combination treatment severely reduced cell growth in a platinum-resistant tumor xenograft model as compared to treatment with cisplatin alone [146]. The combination treatment induced G_2_/M phase arrest and increased expression of *p53* and *p21* as compared to single therapy. Furthermore, cisplatin alone induced only 5–9% of apoptosis, whereas, combinational treatment resulted in a 50–70% induction of apoptosis by modulating Bcl-2 family of proteins [146]. Therefore, the combination of cisplatin with HO-3867 is effective in selective targeting of the platinum-resistant ovarian cancer cells by inhibiting STAT3 activity (Figure 4a).

### 4.2. WNT/β-Catenin Pathway

A large number of reports have shown evidence supporting the involvement of wingless-related integration site (Wnt)/β-catenin in cancer initiation and progression [147,148,149]. One of the main components that drives the Wnt pathway is a frizzled receptor that has been a target for various cancer therapy research [150]. In the presence of the Wnt ligand, the pathway is “on”. In the absence of the ligand or inhibition of frizzled receptors the pathway is turned “off”. Upregulation of the Wnt pathway causes continuous proliferation and can disrupt normal cell behaviour leading to the progression of cancer. The β-catenin and frizzled-receptors are the most studied components of the Wnt pathway, as they accumulate in the nucleus and cytoplasm of ovarian cancer cells [151,152]. Modulation of Wnt pathway has been reported to drive chemoresistance, where β-catenin may function as an oncogene favouring cancer progression [153,154]. Furthermore, inhibition of this pathway in OC has demonstrated an increased response to platinum drugs [155,156].

Sinomenine or SIN (7,8-didehydro-4-hydroxy-3,7-dimethoxy-17-methyl-a, 13a, 14a-morphinan-6-one) (Figure 2f), a compound extracted from Chinese plant *Sinomenium acutum* is known for its therapeutic effects on many diseases, including anti-rheumatic and anti-inflammatory properties [157]. The anti-tumorigenic activity of SIN has been reported in various types of cancers e.g., lung cancer, hepatocellular cancer, and breast cancer [158,159,160]. Although it was known that SIN played a role in reducing tumour proliferation and metastasis in OC, the mechanism was only recently revealed. Li et al. demonstrated that SIN inhibits the expression of oncogenic mini-chromosome maintenance protein 2 (MCM2) and Wnt/β-catenin pathway resulting in tumour growth and metastasis suppression [161]. MCM2, a regulator of DNA replication, is known to be associated with ovarian cancer malignancy. MCM2 expression was found elevated in ovarian tumours that were obtained from over 75 participants when compared to normal tissue [161]. Its knockdown resulted in the degradation of β-catenin, along with downregulating the expression of c-Myc and cyclinD1 (Figure 4B). SIN treatment in cells reduced the expression of MCM2, c-Myc, cyclinD1 and β-catenin, leading to apoptosis. Hence, these data suggest that SIN promotes apoptosis through suppression of the Wnt/β-catenin pathway via MCM2 modulation.

Berbamine (Figure 2g), a product extracted from the plant *Berberis amurensis* has also been shown to inhibit Wnt/β-catenin pathway in ovarian cancer cells [162]. This compound has anti-tumorigenic activity in many cancers and has been recently tested in OC cell lines SKOV3 and ES2 [162,163,164]. Berbamine inhibited cell viability in these cell lines and tumor growth in a SKOV3 subcutaneous xenograft model [162]. Flow cytometric analyses showed that berbamine induced cell cycle arrest at G_0_/G_2_ phase, increasing apoptotic rate as compared to controls. Additionally, western blot analysis showed an increase in cleaved caspase-3, caspase-9, and Bax whereas, Bcl-2 was decreased. This result stipulates that berbamine successfully reduces the concentration of anti-apoptotic proteins in the ovarian cancer cell lines. Lastly, quantitative real-time PCR results supported that berbamine causes inhibition of Wnt/β-catenin signaling. Together, these data showed that inhibition of the Wnt/β-catenin pathway promotes apoptosis. However, these results cannot be verified unless they can be reproduced using xenograft or patient-derived organoids models before advancing to clinical trials.

### 4.3. MET/HGF Pathway

Hepatocyte growth factor receptor (c-MET) is a tyrosine kinase receptor that initiates various cellular processes such as proliferation, cell survival, and angiogenesis (Figure 4C) [165,166,167]. c-MET has been reported to inhibit apoptosis in cancer cells and induce chemotherapy resistance [168]. The over-expression of c-MET is found in 7–27% of EOC [169,170,171,172], and its activation was associated with poor survival in patients with lung, breast, stomach, kidney, and head and neck cancers [169,170,171,172].

Crizotinib (SU11274; Figure 2h) is a c-MET kinase inhibitor that has been tested in both in vitro and in vivo models of OC [173]. It inhibited c-MET phosphorylation thereby downregulating the phosphorylation of downstream proteins, protein kinase B (AKT) and ERK. Amongst 16 normal ovarian, 47 serous carcinoma and 16 ovarian clear cell carcinoma tissues examined, high levels of c-MET were significantly detected in clear cell carcinomas. Treatment with crizotinib decreased cell proliferation and triggered apoptosis in ovarian clear cell carcinomas that expressed elevated c-MET levels. 

Another study examined the effect of crizotinib in 119 human ovarian cancer cells with c-MET overexpression [171], similarly concluded the elevated activity of crizotinib in ovarian cancer cells expressing high c-MET. These data elude to the use of crizotinib in the potential treatment of OC that overexpress c-MET. A recent clinical trial (ClinicalTrials.gov Identifier: NCT02034981) has been initiated to assess the efficacy of crizotinib in cancer patients identified with amplication of crizotinib target genes—anaplastic lymphoma kinase (ALK), MET or ROS proto-oncogene 1 (ROS1) positive (Table 1). However, there were only seven ovarian cancer patients out of 635 with amplified MET expression. Later, other studies suggested that crizotinib is more effective in targeting ALK amplification as compared to MET. Hence the drug has been undergoing clinical trial (ClinicalTrial.gov Identifier: NCT02465060, NCT02568267) to assess the efficacy in patients with ALK and ROS1 amplification (Table 1). However, the results from these clinical trials are still awaiting.

BMS-777607 (Figure 2i) is another small molecule c-MET inhibitor which has shown promising anti-tumorigenic activity against prostate cancer metastasis, as well as being studied in ovarian cancer cells [174,175]. BMS-777607 induced the highest inhibition of cell growth in cells constitutively expressing c-MET, ultimately leading to inhibition of c-MET phosphorylation. This finding was demonstrated in various human ovarian cancer cell lines such as—SKOV3, ES-2, TOV112D, TOV21G, A2780, OVCAR3, OVCAR8 and a human tumor xenograft model in mice [174]. As a result, BMS-777607 treatment increased apoptosis and decreased xenograft tumor mass and invasive properties of the cells [174]. Cabozantinib (XL 184) is also an inhibitor of MET undergoing clinical trials for ovarian cancer treatment (ClinicalTrial.gov Identifier NCT00940225). The results from the first clinical trial appear positive, though the results from another clinical trial are still pending. In addition, MK8033 has also been identified as a novel drug molecule for targeting MET in ovarian cancer cells. This molecule has only been tested in ovarian cancer cell lines and xenograft models, clinical testing will be needed to really understand its usefulness. Nevertheless, these c-MET inhibitors show promising results against ovarian cancer cells, however, the response in long-term use in treatment requires further investigation.

### 4.4. MAPK/ERK Pathway

Mitogen-activated protein kinase (MAPK) plays a crucial role in the survival of cancer cells [176]. Extracellular signal-regulated kinase (ERK) and MAPK pathway are activated upon various conditions such as DNA damage, changes in protein concentrations, stimuli from external factors and intracellular signaling [177]. In addition, minor alteration in genes that regulate cell differentiation, genome stability, and survival can lead to over-activation of this pathway [178]. The mutations in MAPK/ERK pathway have been identified as a promising target in cancer and treatment options are being developed with an aim to alter this tumor signaling pathway that contributes to carcinogenesis. 

Rey et al. generated a human-derived IgM monoclonal antibody (Mab216) that binds to poly *N*-acetyl lactosamine present on hematologic and solid tumors including EOC [179]. Interestingly, this antibody does not bind to any normal tissue other than B-cell, T-cell, and RBCs, thereby causing minimal side-effects [180]. Mab216 leads to deactivation of MAPK/ERK pathway (Figure 4D) thereby inhibiting metastatic and invasive properties of cells. Another study has confirmed the apoptotic effects of Mab216 on ovarian cancer cells [181]. The study claimed that the combination of cisplatin and Mab216 increased the cytotoxicity in EOC. The data suggests that the antigen expressed by EOC is similar to poly *N*-acetyl lactosamine epitome commonly found in B cells. Thus, Mab216 binds to these antigens causing cell apoptosis. The selective inhibitory effects of Mab216 in EOC cells makes it a promising candidate for therapeutic studies.

Flavonoids are another MAPK pathway inhibitor of interest, specifically delphinidin (Figure 2j) and resveratrol [53,182,183]. Flavonoids are polyphenols abundantly found in the human diet in the form of vegetables and fruits. They can be subdivided into 6 categories based on the structural and functional features: anthocyanidins, flavanols, flavanones, flavonols, flavones, and isoflavones [184]. Previous studies have shown that flavonoids were effective in treating osteoporosis, cardiovascular, neurodegenerative diseases, diabetes, and cancer [185]. Later, the role of flavonoids in inhibiting cancer progression was demonstrated in multiple studies. Flavonoids are known to obstruct DNA synthesis causing modulation in the cell survival/proliferation pathways leading to apoptosis in cancer cells [186,187,188,189,190,191,192]. Delphinidin belongs to anthocyanidin family and is present in red cabbage, berries, sweet potatoes and grapes. Lim et al revealed that delphinidin has inhibitory effects on the proliferation of ovarian cancer cells [182]. It reduced SKOV3 cell line proliferation as compared to paclitaxel and had activity against paclitaxel-resistant SKOV3 cells. Delphinidin altered the phosphorylation status of AKT, ribosomal protein S6 kinase β-1 (P70S6K), ribosomal protein S (S6), ERK1/2, and p38. Other studies have shown delphinidin activity in ovarian clear cell carcinoma and the ability to reduce brain-derived neurotrophic factor-induced cell migration and invasion in SKOV3 [193,194]. This drug is yet to be tested in other ovarian cancer cell models including patient-derived cells.

Resveratrol (trans-3,4,5’-trihydroxystilbene) has also been reported to have anti-cancer properties in various human cancers including ovarian [195,196,197,198,199]. Various apoptotic mechanisms of resveratrol have been proposed including phosphorylation of Cdc2-tyrl5 leading to cell cycle arrest in OVCAR3 cells and the downregulation of AKT/ERK pathways causing initiation of apoptosis [198]. However, Lin et. al. suggested that resveratrol exploit ceramide and cyclooxygenase-2 (COX-2) to induce apoptosis in OVCAR3 cells [197]. In resveratrol treated cells, there was an accumulation of COX-2 in the nucleus and Bcl-x short accumulation in the cytosol, while the concentration of COX-1 was unchanged. Additionally, to investigate the claims of MAPK pathways being involved in resveratrol-induced apoptosis, the cells were treated with a combination of resveratrol and MEK inhibitor PD98059 or p38 kinase inhibitor SB203580. The results showed a progressive decrease in the levels of COX-2 in cells treated with resveratrol and MEK inhibitor and revealed that activation of ERK1/2 was consistent with a decrease in COX-2 levels in the nucleus. This indicates the important role of MAPK/ERK pathway in resveratrol-induced apoptosis. However, more studies using patient-derived cells may be required to verify these findings.

### 4.5. PI3K/AKT/mTOR Pathway

Phosphatidylinositol 3-kinase (PI3K)/phosphatase and tensin homologue (PTEN)/v-AKT murine thymoma viral oncogene homologue (AKT)/mammalian target of rapamycin (mTOR) pathway is the control house of growth signal transmissions [200]. The loss of *PTEN* along with other mutations causes over-expression of the PI3K/AKT pathway resulting in uncontrolled cell-cycle progression, reduced apoptosis, and increased metastasis [201,202]. Enhanced expression of PI3K/AKT is also recognized as a hallmark of various cancers including ovarian [203]. Previous studies have also shown that the inhibition of PI3K/AKT pathway leads to apoptosis of tumor cells [204,205]. Thus, this pathway has been a target for cancer treatment and new molecules are being investigated.

Honig et. al. studied the role of D-116883, an oral PI3K inhibitor, to induce apoptosis in platinum-resistant and platinum-sensitive cell lines of A2780, as well as other ovarian cancer cell lines including OAW42, SKOV3 and human peripheral blood lymphocytes (PBL) [206]. D-116883 inhibited the phosphorylation of AKT post-treatment leading to cell cycle arrest in G0 phase and eventually causing apoptosis in human ovarian cancer cells [206]. This study shows promising results in targeting platinum-resistant ovarian cancer cell lines. Hence, it should be further investigated in better clinical models such as patient-derived organoids and xenograft models.

Alisertib (ALS) is an Aurora kinase inhibitor that has been extensively studied for its anti-carcinogenic properties against ovarian cancer cells [207,208]. In SKOV3 and OVCAR4 cell models, ALS caused G_2_/M phase arrest followed by mitochondrial-mediated apoptosis, through inhibition of PI3K/AKT/mTOR and p38 MAPK pathways. Currently, a number of clinical trials are examining ALS in combination with chemotherapy in recurrent ovarian cancer patients (Table 1) [208,209]. A clinical trial performed using MLN8237, an alisterib, showed modest anti-tumor effects and promising response in patients with chemoresistance [210]. Following the success of this clinical trial, MLN8237 has entered phase II/III trial where it will be administered in combination with paclitaxel in ovarian cancer patients.

An important component of AKT/nuclear factor kappa-light-chain-enhancer of activated B cells (NF-κB) pathway is a chemokine, C-C motif ligand 5 (CCL5) which regulates metastasis and migration of tumor cells [211]. A recent study has reported that CCL5 is elevated in ovarian cancer cells and plays a role in inhibiting apoptosis [212]. In this study, treatment with cordycepin reduced cell viability in ovarian cancer cell lines SKOV3, MDAH-2774, and OVCAR-3 while also influenced cellular apoptosis through downregulation of AKT/NF-κB pathway and upregulation of caspase-3. Additionally, cordycepin reduced tumor cell migration in the cell lines tested. These data suggest that further investigations using in vivo models and patient-derived cancer cells are required to test the efficacy and safety of D-116883 and cordycepin.

## 5. Conclusions

Apoptosis is a critical process to initiate in a cancer cell, but it involves various complex pathways and regulators. Cancer cells have been known to exploit this process to evade cell death. Although many targets in ovarian cancer have been identified, the search for a drug that demonstrates robust capacity to promote cancer cell death is ongoing. Thus, various studies have been focused on reversing anti-apoptotic mechanisms. To date, these approaches include (i) direct targeting of molecules involved in apoptotic pathway that undergo modification in cancer cells; (ii) indirect targeting of molecules that lead to inhibition of apoptotic process, and (iii) targeting of cell signaling pathways that allow cancer cells to resist death. Drugs such as TW-37, Navitoclax, birinapant and DEBIO-1143 have been found useful in mimicking or targeting apoptotic related proteins. Indirect inhibitors of apoptosis such as DPI, PRIMA-1^MET^, and INCB054329 showed promise in preliminary ovarian cancer cell testing. Apoptosis can also be achieved by obstructing cell signaling pathways such as JAK/STAT3, Wnt/β-Catenin, MET/ Hepatocyte growth factor (HGF), MAPK/ERK, and PI3K/AKT/mTOR. Various studies have investigated small molecule inhibitors that act on these signaling pathways such as HO3867, Sinomenine, SU11274, BMS-777607, Mab216, and Delphinidin.

Overall, from these previous studies, it can be concluded that apoptosis can be effectively induced through direct and indirect targeting in ovarian cancer cells with promising results. Bcl-mediated apoptosis has been one of the most studied cellular pathways due to the involvement of many components inter-dependent for inducing apoptosis in a cell. BH3 mimetics have gained commendable response in clinical trials. Importantly, the efficacy of these therapies may be further improved with the overexpression of the relevant apoptotic molecules in the tumors. Another most exploited pathways in ovarian cancer therapy relates to p53 and PARP that have been an indicator for platinum-resistant disease. In the cellular signaling pathway, there are multiple components that have not been fully understood. Most of these components are involved in more than one cellular process that could either be essential for apoptosis or lead to a different form of cell death. Thus, inhibitors of cellular pathways can cause non-specific targeting of one or many molecules, ultimately causing apoptosis via another mechanism. For example, crizotinib was introduced as a MET inhibitor to target MET/HGF pathway. However, upon further investigations, it became apparent that crizotinib is an effective drug for targeting ALK and ROS1 in addition to MET. Since then, it is being tested as a multi-target drug. Therefore, target specificity should be carefully considered in all circumstances.

Lastly, although some of the drugs mentioned in this review have shown commendable response in vitro treatment or clinical trials, better results can be achieved by in-depth investigation on controlling toxicity and enhancing the efficacy of these drugs. Furthermore, additional studies focused on investigating cross-talk between cellular pathways within the tumour microenvironment and their response to drugs is required. This will give a better understanding of mechanisms adopted by cancer cells in developing drug-resistance. Future development and research into apoptotic inducers and the forthcoming drugs may prove useful in limiting cancer progression.

## Figures and Tables

**Table 1:** Tabular representation of drugs and their corresponding clinical trial information.

Drug	Target	Clinical Trial ID	Number of Patients	Phase	Result
Birinapant	IAPs	NCT01681368	11	2	SD = 2 (18.2 %) upto 4-5 months CR = 0 PR = 0
Birinapant	IAPs	NCT01940172	18	1	PR = 1 SD = 4
DEBIO1143	IAPs	NCT01930292	11 out of 31 with ovarian cancer	1	PR = 4 (36.4%) SD = 4 (36.4%) PD = 3 (27.2%) Terminated
PRIMA-1MET	P53	NCT02098343	200	1 & 2	Ongoing
Alisertib	Aurora kinase (PI3K/Akt/mTOR pathway)	NCT01091428	191 and 142 respectively	1 & 2	median PFS increased by 2 months
Alisertib	Aurora kinase (PI3K/Akt/mTOR pathway)	NCT00853307	31	2	CR = 3 (10%) PR = 4 (31%) SD = 7 (23%) PD = 12 (40%) NE = 5 (16.1%)
Crizotinib	MET/ALK/ROS1	NCT02465060	7/635 with MET amplification (ovarian cancer)	2	Ongoing
Crizotinib	MET/ALK/ROS1	NCT02568267	300 patients with different types of cancer	2	Ongoing
Cabozantinib	MET	NCT00940225	70	2	PR = 14 (20%) OS = 50% at 12 weeks PFS= 5.9 months
Cabozantinib	MET	NCT02315430	13	2	Results awaited

CR = complete response, PR = partial response, SD = stable disease, PD = progressive disease, OS = overall survival, PFS = Platinum free survival, and NE = inevaluable, IAPs = inhibitor of apoptotic proteins, PI3K = phosphatidylinositol 3-kinase, mTOR = mammalian target of rapamycin, MET = mesenchymal-epithelial transition factor, ALK = anaplastic lymphoma kinase, ROS1 = ROS proto-oncogene 1.
